# Integrative Histologic and Bioinformatics Analysis of BIRC5/Survivin Expression in Oral Squamous Cell Carcinoma

**DOI:** 10.3390/ijms19092664

**Published:** 2018-09-08

**Authors:** Giuseppe Troiano, Agostino Guida, Gabriella Aquino, Gerardo Botti, Nunzia Simona Losito, Silvana Papagerakis, Maria Carmela Pedicillo, Franco Ionna, Francesco Longo, Monica Cantile, Antonio Pennella, Lucio Lo Russo, Giovanni Di Gioia, Maria Addolorata Mariggiò, Lorenzo Lo Muzio, Giuseppe Pannone

**Affiliations:** 1 Department of Clinical and Experimental Medicine, University of Foggia, Via Rovelli 50, Foggia 71122, Italy; giuseppe.troiano@unifg.it (G.T.); mariacarmela.pedicillo@unifg.it (M.C.P.); antonio.pennella@unifg.it (A.P.); Lucio.lorusso@unifg.it (L.L.R.); digioia-giovanni@outlook.it (G.D.G.); lorenzo.lomuzio@unifg.it (L.L.M.); giuseppe.pannone@unifg.it (G.P.); 2 Maxillofacial and ENT Surgery Department, Istituto Nazionale per lo Studio e la Cura dei Tumori, Fondazione G. Pascale, IRCCS, Naples 80131, Italy; agoguida@gmail.com (A.G.); f.ionna@istitutotumori.na.it (F.I.); f.longo@istitutotumori.na.it (F.L.); 3 Pathology Unit, Istituto Nazionale per lo Studio e la Cura dei Tumori, Fondazione G. Pascale, IRCCS, Naples 80131, Italy; gabryaquino@gmail.com (G.A.); g.botti@istitutotumori.na.it (G.B.); s.losito@istitutotumori.na.it (N.S.L.); 4 Department of Surgery, Cancer Research Cluster, Room 4D10.2, Health Sciences Building, Saskatchewan University, Saskatoon, SKS7N5E5, Canada; silvana.papagerakis@usask.ca; 5 Department of Biomedical Sciences and Human Oncology, University of Bari. Piazza Giulio Cesare 11, Bari 70124, Italy; mariaaddolorata.mariggio@uniba.it (M.A.M.)

**Keywords:** Survivin, BIRC5, immunohistochemistry, oral cancer, bioinformatic, TCGA

## Abstract

Survivin is a well-known protein involved in the inhibition of apoptosis in many different cancer types. The aim of this study was to perform an integrated bioinformatic and histologic analysis in order to study the expression and prognostic role of Survivin and its related gene *BIRC5* in oral cancer. Publicly available databases were accessed via Gene Expression Omnibus and Oncomine, in addition raw data from The Cancer Genome Atlas (TCGA) were also obtained in order to analyze the rate of gene mutation, expression and methylation in patients with oral squamous cells carcinoma (OSCC). Immunohistochemistry (IHC) was also performed in order to evaluate the nuclear and cytoplasmic expression of Survivin and their correlation with cell proliferation in samples from OSCC patients. Results of this study revealed that Survivin is rarely mutated in OSCC samples and upregulated when compared to non-cancerous tissue. A negative correlation between the methylation of the island cg25986496 and BIRC5 mRNA expression was detected from TCGA data. IHC staining revealed that cytoplasmic (and not nuclear) expression of Survivin is associated with poor overall survival in OSCC patients, while the nuclear expression correlates with higher proliferation rate. In addition, data from TCGA database revealed that BIRC5 gene expression is an independent prognostic factor for OSCC patients.

## 1. Introduction

Over 500,000 new cases of head and neck squamous cell carcinoma (HNSCC) are reported annually worldwide [1]. Different subsites show various frequencies and environmental causes in world regions; in developed countries, approximately 75% of the oral cavity, larynx and pharynx cancers are attributable to tobacco smoking and alcohol consumption [2]. Oral squamous cell carcinoma (OSCC) is the most common subtype of HNSCC, accounting for more than 200,000 new cancer cases every year globally [3,4]. OSCC may appear in any location of oral cavity, although there are some anatomic subsites in which it is more commonly found: the anterior two-thirds of the tongue and the floor of the mouth [5,6,7,8,9]. Other subsites that may be involved are: buccal mucosa, retromolar area, gum, lip, soft palate and, less frequently, back of the tongue and hard palate. The etiology of OSCC is multifactorial; the most known risk factors are tobacco, excessive consumption of alcohol [10] and betel quid usage [11]: these factors can act separately or synergistically [12]. Other factors such as human papillomavirus (HPV) infection may also be involved [13], especially in the oro-pharynx. The overall five-year survival rate for OSCC is around 50–60%, generally; such poor prognosis for OSCC is mostly accounted for by presentation at a late stage of the disease [14]. Some studies show that patients usually delay seeking professional advice on average for periods up to three months after having become aware of any oral symptom that could be linked to oral cancer [15]. Since cancer is still the second cause of death globally, after cardio-vascular disease, a lot of attention has been paid to its biomolecular mechanisms. Increasing evidence indicates that Survivin, a member of the inhibitor of apoptosis (IAP) protein family, is not only an essential protein molecule for apoptotic inhibition and regulation of mitosis, but it also plays a role in certain physiological processes, as well as in pathological conditions such as carcinogenesis in many human organs/cells [16,17].

It has been shown in various types of cancer that the transcriptional level of Survivin correlates with a more aggressive disease progression and poor clinical outcomes. In some types of cancers, the overexpression of Survivin may lead to overcoming the cell cycle checkpoints and thus, facilitate an aberrant progression of transformed cells through mitosis [18]. Survivin is usually overexpressed at G2/M phase, while its expression declines rapidly in G1 phase of the cell cycle. The baculoviral IAP repeat containing 5 (BIRC5) gene encoding human Survivin was cloned by Ambrosini et al. [19], it is 14.7 kb long, located near the telomeric end of chromosome 17 and encodes for the production of the wild-type Survivin protein [20]. Survivin is the smallest member of the IAP family and all its isoforms are characterized by containing only one of the characteristic N-terminal BIR (Baculovirus IAP Repeats) domains [17,21]. In order to help to organize, analyze, understand, visualize and store all information associated with biological macromolecules, the application of computational tools is becoming extremely helpful, giving birth to bioinformatics [22].

Bioinformatics has different aims: first, it allows researchers to organize data in an easier manner, to access them and add new entries as they are generated; the second purpose aims to develop tools and resources that help researchers in the process of data analysis; the third aim is to use these tools to cross reference data analysis and results interpretation in order to explain such findings from a biological point of view. Different types of data can be accessed via bioinformatics, including sequencing and expression results on a wide scale, allowing in this way to analyze results on a whole-genome and whole-exome level [23,24,25,26,27].

One of the advantages of performing bioinformatic analysis is the possibility to group together data on the basis of biological similarities accessing different sources of research [28]. Some large cancer projects, like The Cancer Genome Atlas (TCGA), have been performed, which aim to generate genomic and molecular profiling datasets and make them available to the scientific community for further analysis [29]. TCGA generated molecular profile data at the levels of gene expression, protein expression, DNA copy, DNA methylation, and somatic mutation [30]. The use of these databases allows users to perform integrated well-standardized analysis on various type of cancer, paving the way for studies aiming to integrate data from single institutions with that of multi-institutional databases [31].

The aim of this study was to perform an integrated bioinformatic and histologic analysis in order to study the expression of Survivin and its related gene *BIRC5* in OSCC. The bioinformatics analysis focused on genetic mutations, mRNA expression, methylation and gene network. In addition, immunohistochemistry analysis from a single institution database was performed in order to study the prognostic significance of cytoplasmic and nuclear expression of Survivin in OSCC.

## 2. Results

### 2.1. Bioinformatic Analyses

**Figure 1:**
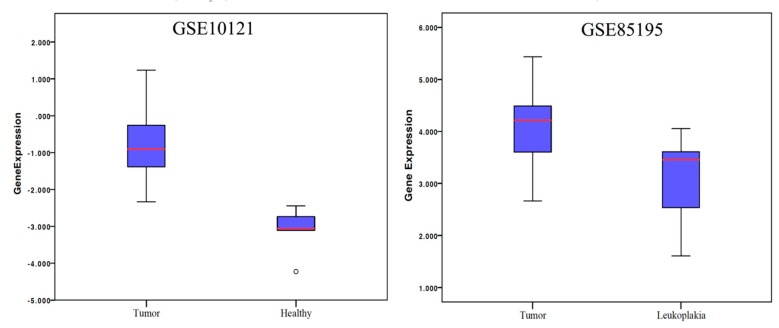
Analysis of previously published data comparing BIRC5 mRNA expression in cancer vs. non-cancer samples.

**Figure 2:**
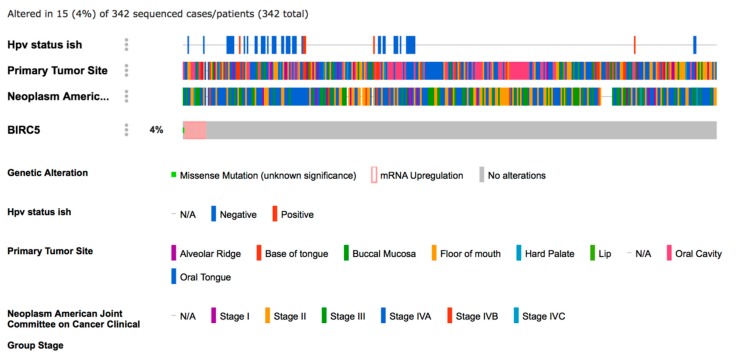
Analysis of OSCC patients included in the TCGA database by means of cBIOportal (http://www.cbioportal.org/) [32,33].

**Figure 3:**
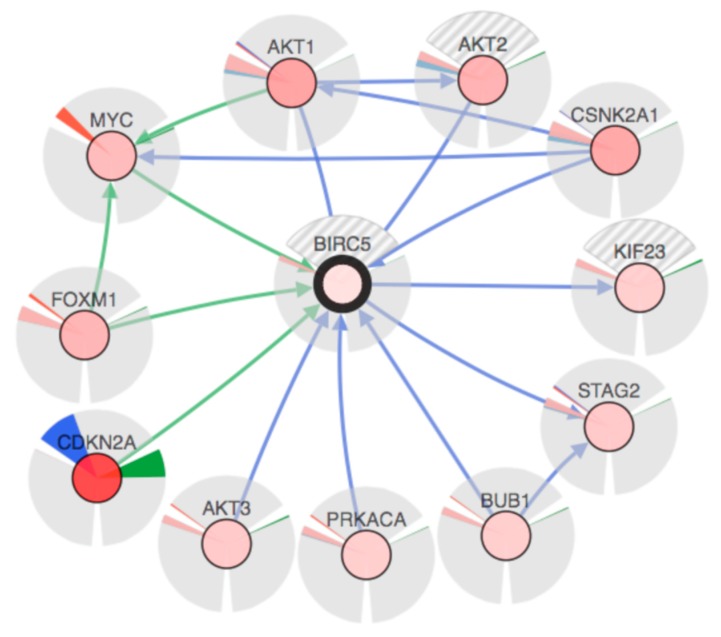
Network involved in the expression of the *BIRC5* gene. Blue lines indicate genes controlling the state change of those genes to which the arrows are pointing; while the green lines indicate genes controlling the expression of those genes to which the arrows are pointing. Each gene is represented by a colored nucleus, indicating its overall alteration in the *BIRC5* expression (the stronger the color intensity, the greater the alteration) surrounded by three areas: one filled with the color green, indicating how much the gene is mutated; the second one filled by both blue and red colors, indicating respectively the amount of homozygous deletion and the amplification of the gene; the third one filled by both pink and light blue colors, indicating respectively the upregulation and downregulation values of the gene. Where one or more areas are filled by grey and white stripes, data are missing.

Results of the comparative analysis between OSCC samples and non-cancer tissues revealed that the mRNA expression of BIRC5 was higher in the cancer samples compared both to leukoplakia samples and to normal tissue in healthy (non-cancerous) patients (Figure 1). The rate of BIRC5 mutations in 342 OSCC samples included in TCGA database revealed that only one (0.29%) sample showed missense mutation, while mRNA upregulation was recorded in 14 (4.09%) samples (Figure 2). Data from in situ hybridization (ISH) revealed that five samples (1.46%) were HPV positive. In addition, crossing data by primary subsite onset revealed that four out of five of these tumors were located at the base of the tongue, while for the remaining one the subsite of origin was unknown (Figure 2). Analysis of the network revealed that CDKN2a, MYC and FOXM1 control the expression of BIRC5, while AKT1-3, PRCACA, BUB1 and CSNK2A1 control a reaction that changes the state of the Survivin protein (Figure 3). Correlations analysis between BIRC5 mRNAs expression, methylation and clinicopathologic parameters of patients with OSCC revealed a statistically significant inverse correlation between the methylation of the island cg25986496 and the mRNA expression of BIRC5 (ρ = −0.125), in addition mRNA expression correlated with the stage of the disease (ρ = 0.133) (Table 1). Cox-regression survival multivariate analysis revealed that BIRC5 mRNA expression was an independent prognostic biomarker of overall survival (*p*-value = 0.008) (Table 2), while the methylation rate of the island cg25986496 showed *p*-values close to the significance (*p*-value = 0.068) for disease-free survival.

### 2.2. Tissue Micro Array (TMA) Immunohistochemistry (IHC) Analysis

**Figure 4:**
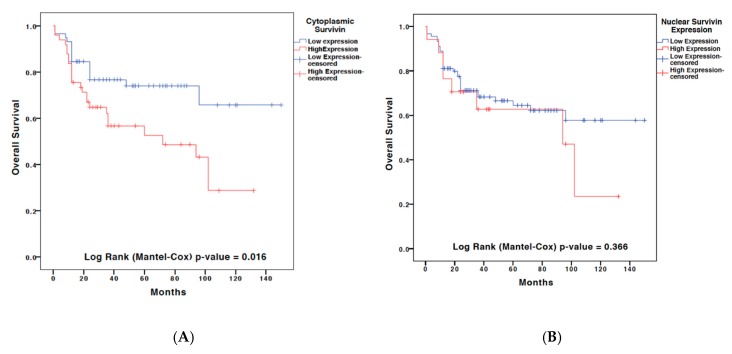
Kaplan–Meier curves of overall survival for the immunohistochemical expression of (**A**) cytoplasmic or (**B**) nuclear cellular localization of Survivin in the authors’ own database.

Cytoplasmic and nuclear staining of Survivin were performed through IHC analysis on a TMA comprising 107 samples of OSCC patients admitted to the National Cancer Institute “Giovanni Pascale” between 1997 and 2008. The clinical and pathological information of patients included in the TMA have been summarized in Table 3. The immunostaining findings were expressed as a percentage of positivity of the cytoplasmic or nuclear staining of Survivin-positive cells. A threshold of 60% of positivity was chosen to subcategorize patients relative to low/high expression of Survivin. Pearson analysis revealed a direct correlation between the cytoplasmic and nuclear expression of Survivin (ρ = 0.319). In addition, a direct correlation with the histological grade of the tumor was also recorded for both nuclear (ρ = 0.215) and cytoplasmic (ρ = 0.218) expression. Both Kaplan–Meier (univariate) and Cox regression (multivariate) analysis revealed that only the cytoplasmic expression of Survivin was an independent prognostic factor of overall survival in OSCC (Figure 4 and Table 4).

IHC of leukoplakia (*N* = 10 cases) and normal mucosa (*N* = 12 cases) samples from healthy (non-cancerous patients) revealed that Survivin expression was almost exclusively nuclear and confined to the basal third of the epithelium (Supplementary Materials). Particulary, the average percentage of Survivin expression was 8.3% in the leukoplakia samples, versus 0.54% in the normal mucosa.

## 3. Discussion

Survivin is a well-known protein that belongs to the family of the inhibitor of apoptosis proteins (IAP) family. It is encoded by the *BIRC5* gene located on the chromosome 17q25 [19].To our knowledge, only one meta-analysis has been previously published by pooling data from various studies evaluating the significance of Survivin (at either mRNA or protein levels) as a prognostic factor in OSCC patients. Results of such meta-analysis were controversial encouraging the development of further cohort studies on the topic [34]. In this current study, we decided to perform an integrated analysis of BIRC5/Survivin expression using both IHC analysis on authors’ institutional databases and a bioinformatics analysis on publicly available databases. Although a direct comparison between these two databases (GSE10121 and GSE85195) was not possible, our comparative analysis indicated that the BIRC5 mRNA was upregulated in OSCC compared both to leukoplakia and oral normal tissue (Figure 1). In addition, the presence/absence of mutations of the *BIRC5* gene was analyzed revealing that this gene is very rarely mutated in OSCC. Furthermore, already published databases were accessed through Oncomine and GEO2r in order to compare the expression of BIRC5 mRNA in OSCC, precancerous tissue and normal tissue from healthy non-cancerous patients. The bioinformatic analysis also allowed us to evaluate a novel direction relevant to the OSCC topic, that is the low frequency of HPV-positive OSCC tumors with respect to other sites in the head and neck area. The results obtained from the analysis of the TCGA database are in agreement with previous studies from other cohorts, which revealed a low rate of HPV positive tumors in the oral cavity, located particularly at the base of the tongue [35,36,37,38]. One of the main challenges in the OSCC study through online tools is that TCGA database includes cases from the whole head and neck area, which makes it difficult to perform subgroup analysis targeting only the OSCC patients. For such reasons, we decided to access the raw data from the TCGA database and manually select cases by the primary tumor anatomical site, which allowed us to identify 342 OSCC patients which we included in this current analysis. 

We then analyzed this cohort composed of these 342 OSCC patients and their respective available data of mRNA expression and methylation rate through cBIOportal. The multivariate analysis of this data revealed that BIRC5 mRNA expression is an independent prognostic factor in OSCC and correlates with tumor stage. These findings are in accordance with what was previously reported in the literature [39,40]. In addition, we found a group of genes that seem to be highly involved in BIRC5 expression: *AKT1*, *AKT2*, *AKT3*, *BUB1*, *CDKN2A*, *CSKN2A1*, *FOXM1*, *KIF23*, *MYC*, *PRKACA*, *STAG2*. In Figure 3 it can be noticed that the mostly altered gene is *CDKN2A*, which is also showing the highest levels of mutation and homozygous deletion. Only *CDKN2A*, *MYC* and *FOXM1* are responsible for the control of the BIRC5 expression, while the remaining genes control the genetic alterations of BIRC5. It is critical to note that *AKT1* and *FOXM1* also control the expression of *MYC*, while *CSNK2A1* controls its state of change (i.e. genetic alterations). *CSNK2A1* also controls the genetic alterations of both *MYC* and *AKT1*, which in turns control the state of change of *AKT2*. A statistically significant inverse correlation was found between the methylation rate reported on cBioPortal and BIRC5 mRNA expression (ρ = −0.125). It is important to highlight only methylation data from the probe with the strongest negative correlation between the methylation signal and the respective gene expression were included in the cBioPortal data for genes with multiple probes. Hence, we decided to download all the raw data from the TCGA database in order to find out which was the probe involved in the correlation with BIRC5 expression. By matching data between cBioPortal and TCGA, we found that the correlation was related to the island cg25986496. The possibility that the gene expression may be regulated by an epigenetic mechanism has already been reported [41]. Further functional studies on cell lines are needed to confirm this association between the methylation of the island cg25986496 and BIRC5 mRNA expression.

**Figure 5:**
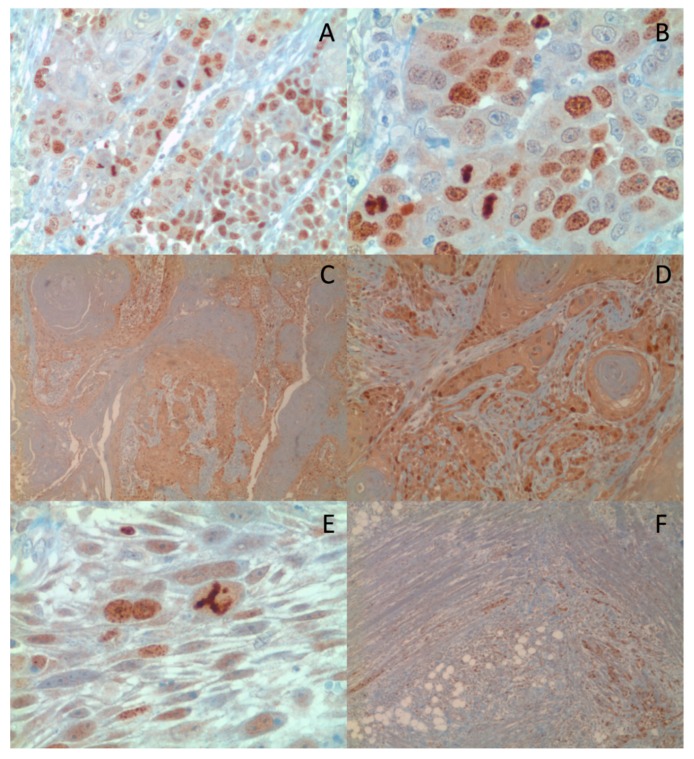
Nuclear (**A** 10x, **B** 20x) and Cytoplasmic (**C** 4x, **D** 10x) expression of Survivin by IHC. Survivin was also expressed in cases showing aberrant mitosis (**E** 40x) and muscular invasion (**F** 4x).

To address the discordance among various studies regarding prognostic value of the Survivin, we extended our analysis and assessed its intracellular distribution [34,42,43,44]. Thus, we evaluated the Survivin cytoplasmic and nuclear localization in a TMA composed of 107 OSCC cases; in addition, 12 cases of leukoplakia and 10 cases of normal mucosa samples from healthy (non-cancerous) patients were comparatively analyzed. The Survivin “cytoplasmic” or “nuclear” IHC staining extend was subsequently scored as high versus low in the analyzed sample, based on a 60% cut-off score of positivity; no difference was noted in the intensity of the Survivin expression, thus the staining intensity was not scored (Figure 5). Our IHC analysis has shown that Survivin was only weakly expressed in the basal third of oral mucosa in both leukoplakia and normal oral mucosa samples. Furthermore, in contrast to what was previously reported [42], Survivin was found predominantly expressed in the nuclei, while a weak cytoplasmic expression was only noted in one case of leukoplakia. Staining for Ki67 was also performed in the same TMA in order to investigate the correlation of the subcellular localization with cells proliferation. This analysis revealed that the nuclear localization of Survivin was correlated with a higher cell proliferation (quantified as percentage of Ki-67 expression). These findings are in alignment with other previously published studies, which reported that patients with higher nuclear expression of Survivin had a better response to radiotherapy [45]. Furthermore, our study found that both the cytoplasmic and nuclear expression of Survivin correlated with tumor grade, while no correlation with the tumor stage was detected. In order to study whether the subcellular location of Survivin correlated with OSCC prognosis, we performed a multivariate analysis taking into account the OSCC tumor stage, grade, patient sex and age and the Survivin cytoplasmic or nuclear expression levels. Results of this analysis revealed that the cytoplasmic localization of Survivin (*HR* = 2.040) and the tumor stage (*HR* = 4.938) were independent prognostic factors in OSCC. Results of this TMA analysis revealed that the cytoplasmic localization of Survivin (*HR* = 2.040) and the tumor stage (*HR* = 4.938) were independent prognostic factors in OSCC. These data are in disagreement with other papers [42,43,46], and in accordance with what was originally reported by our group in a series of OSCCs using whole section specimens [21]. The discordance with previously published studies could be due to the sensitivity of the different methods of detection that were employed, a more homogeneous and larger cohort of cases (the TMA included 107 pathologists-selected OSCC positive core-biopsies, in their vast majority with higher histological grade and advanced tumor stage), and most importantly that in this study we performed a multivariate analysis adjusting for well-known covariates. Overall, our results are in accordance with previous studies, which indicate that the Survivin cytoplasmic expression is correlated with increased tumor aggressiveness and a lower positive response to radiotherapy treatment in OSCC patients [47,48,49,50].

In conclusion, it is imperative that the subcellular analysis of Survivin expression will be further evaluated through additional well-standardized cohort studies, adjusted for other prognostic covariates, in order to better clarify its role in the prognosis prediction of OSCC patients.

## 4. Materials and Methods

### 4.1. Patients Database

All OSCC patients from the authors institutional database had been treated, not consequentially, at the Istituto Nazionale Tumori “IRCCS—Fondazione G. Pascale”, Naples, Italy, with therapeutic intent for their cancer, according to National Comprehensive Cancer Network (NCCN) guidelines, not undergoing experimental procedures. Informed consent was obtained from all patients, after careful explanations, authorizing re-examination of specimens of biological samples for research purposes, as approved by our Institute in the Resolution of the Extraordinary Commissioner; number: 15, date: 15 January 2016, establishing and regulating our Biobank.

### 4.2. Comparison of BIRC5 Gene Expression between Tumor vs. Non-Tumor Samples

The expression level of the BIRC5 mRNA in OSCC samples compared to normal tissue was analyzed through Oncomine gene expression array datasets (https://www.oncomine.org/). In addition, the gene expression profiles of two published databases (GSE85195 and GSE10121) were downloaded from Gene Expression Omnibus (GEO) using the GEO2R platform (https://www.ncbi.nlm.nih.gov/geo/geo2r/). In the GSE85195 database, data for gene expression profiling of Oral Leukoplakia (OL) and Early Stage OSCC were available [51], while in GSE10121 a comparison between primary OSCCs and oral mucosa from healthy,non-cancerous patients was performed [52].

### 4.3. Analysis of BIRC5 Mutations, Methylation and Associated Network in OSCC from The Cancer Genome Atlas (TCGA) Database

Clinical data from TCGA database for patients with HNSCC were downloaded using the software TCGA2BED [53]. The data were catalogued in Microsoft Excel and manually checked on the basis of the primary site of tumor onset in order to exclude non-OSCC patients. Hence, the ID of OSCC patients in TCGA were manually entered in the cBioPortal for Cancer Genomics (http://www.cbioportal.org) in order to analyze the presence of mutations and investigate the associated network of BIRC5. Data for TCGA methylation derived from Human Methylation-450 Bead Chip assay and data of BIRC5 mRNA expression obtained by RNA-sequencing V2 RSEM (Illumina RNA Sequencing version 2) were also downloaded from cBioPortal. To note, for genes with multiple CpG-Islands, as BIRC5, only methylation data from the probe with the strongest negative correlation between the methylation signal and the gene expression were available (http://www.cbioportal.org/) [32,33]. All the raw data obtained from TCGA and GEO databases were entered in SPSS 21.0 in order to perform statistical analyses of correlation with outcome and survival among different groups.

### 4.4. Analysis of Survivin Expression in a Tissue Micro Array of OSCC, Leukoplakia and Healthy Mucosa Samples

All patients had provided written informed consent for the analysis of samples according to the institutional regulations and the study was approved by the ethics committee of the National Cancer Institute “Giovanni Pascale” and the resulting biobank collection of the analyzed specimens was registered as “Bio-Banca Istituzionale BBI” Deliberation (Number: 15, Date: 15 January 2016). For the analysis of Survivin expression, a tissue microarray (TMA) including pathologists-selected tumor core-biopsies from 107 OSCC cases; these cases were part of a larger TMA of 120 cases OSCC our group previously published [54]. The source paraffin blocks were cored and 0.6 mm cores (area: 0.28 mm^2^) transferred to the recipient master block using Galileo TMA CK 3500 Tissue Microarrayer (ISE TMA Software, Integrated System Engineering, Milan, Italy). Four cores from different areas of the same tissue block were arrayed for each case. All the donor cores were formatted into one recipient block. Hematoxylin and eosin (H&E) staining of a 4-µm of the analysed TMA was used to verify the integrity of all samples. In addition, 12 cases of leukoplakia and 10 cases of normal oral mucosa samples from healthy non-cancerous patients were enrolled as negative controls. Expression of Survivin was identified using a rabbit polyclonal antibody supplied by NOVUS (catalog number NB500-201 NOVUS Biologicals, Littleton, CO, USA) raised against full-length recombinant Survivin; the KI-67 rabbit monoclonal antibody supplied by Roche (Ventana-Roche, Monza, Italy) was used to assess the cell proliferation. Primary Abs were revealed by automated staining device (Ventana Benchmark) using standard linked strepatavidin-biotin horseradish peroxidase technique (LSAB-HRP). Immune-stained cells were detected in 4 high-power fields (HPFs) under an optical microscope (OLYMPUS BX53, at x200). Immune-stained spots were acquired by digital camera and analyzed by ISE TMA Software (Integrated System Engineering), and Cellsens V1.9^®^ Olympus image analysis software.

## Figures and Tables

**Table 1:** Pearson’s correlation of oral squamous cell carcinoma (OSCC) patients in the The Cancer Genome Atals (TCGA) database. Methylation rate refers to the island cg25986496, which was the probe with the strongest negative correlation to BIRC5 expression. * *p*-value lower than 0.05.

Variable	mRNA Expression	Methylation	Age	Stage	Grade
mRNA expression	ρ = 1	ρ = −0.125	ρ = 0.025	ρ = 0.137	ρ = 0.023
	*p*-value = 1	*p*-value = 0.021 *	*p*-value = 0.644	*p*-value = 0.015 *	*p*-value = 0.670
Methylation		ρ = 1	ρ = 0.085	ρ = 0.019	ρ = −0.099
		*p*-value = 1	*p*-value = 0.119	*p*-value = 0.735	*p*-value = 0.070
Age			ρ = 1	ρ = −0.099	ρ = 0.118
			*p*-value = 1	*p*-value = 0.083	*p*-value = 0.033 *
Stage				ρ = 1	ρ = −0.094
				*p*-value = 1	*p*-value = 0.104
Grade					ρ = 1
					*p*-value = 1

**Table 2:** Cox regression analysis of patients included in the TCGA database. * *p*-value lower than 0.05.

Variable	Overall Survival		Disease Free Survival	
	Hazard Ratio	*p*-Value	Hazard Ratio	*p*-Value
mRNA expression	1.182	0.008 *	0.906	0.653
Methylation rate	2.350	0.577	0.004	0.068
Grade	0.779	0.209	0.799	0.657
Stage	0.965	0.637	1.806	0.021 *
Age	0.996	0.474	1.007	0.694
Gender	0.997	0.988	0.574	0.259

**Table 3:** Clinicopathologic features of the OSCC patients in the authors’ institutional database.

ClinicoPathological Parameter	Groups	Number
Age	≥65 years old	63/107 (58.9%)
	<65 years old	44/107 (41.1%)
Gender	Male	76/107 (71%)
	Female	31/107 (29%)
Grade	G1	21/107 (19.6%)
	G2/G3	86/107 (90.4%)
Stage	St1/St2	39/107 (36.4%)
	St3/St4	68/107 (63.6%)
Subsite Involved	Tongue	64/107 (59.8%)
	Others sites	43/107 (40.2%)

**Table 4:** Multivariate analysis of cytoplasmic and nuclear expression of Survivin as indicator of poor overall survival in the OSCC patients included in this study (authors’ institutional database) (Note: * indicates statistically significance with *p* < 0.05).

Clinicopathologic Factor	Overall Survival	
	Hazard Ratio	*p*-Value
Cytoplasmic Survivin	2.040	0.045 *
Nuclear Survivin	0.858	0.726
Grade	1.961	0.220
Stage	4.938	0.001 *
Age	0.895	0.734
Gender	0.781	0.490

## Supplementary Materials

The following are available online at http://www.mdpi.com/1422-0067/19/9/2664/s1.
