# Supplementary material for: Integrative Histologic and Bioinformatics Analysis of BIRC5/Survivin Expression in Oral Squamous Cell Carcinoma
Source: Int J Mol Sci. 2018 Sep 8;19(9):2664. doi: 10.3390/ijms19092664 (PMC6174346; doi:10.3390/ijms19092664)
Supplement: Supplementary file 1 [file ijms-19-02664-s001.pdf]

	Cytoplasmic Expression	Nuclear Expression	Ki-67 Expression	Grade	Stage	Age
Cytoplasmic Expression	ϱ = 1 *p*-value= 1	ϱ = 0.319 * *p*-value= 0.001 *	ϱ = 0.199 *p*-value= 0.644	ϱ = 0.218 * *p*-value= 0.024 *	ϱ = 0.072 *p*-value= 0.458	ϱ = 0.044 *p*-value= 0.654
Nuclear Expression		ϱ = 1 *p*-value= 1	ϱ = 0.309 * *p*-value=0.012*	ϱ = 0.215 * *p*-value= 0.026 *	ϱ = 0.064 *p*-value= 0.516	ϱ = -0.052 *p*-value= 0.592
Ki-67 expression			ϱ = 1 *p*-value= 1	ϱ = 0.196 *p*-value= 0.115	ϱ = 0.008 *p*-value= 0.949	ϱ = 0.117 *p*-value=0.35
Grade				ϱ = 1 *p*-value= 1	ϱ = 0.066 *p*-value= 0.501	ϱ = 0.113 *p*-value= 0.246
Stage					ϱ = 1 *p*-value= 1	ϱ = -0.120 *p*-value= 0.219
Age						ϱ = 1 *p*-value= 1

Supplemental Materials: Pearson's correlation of OSCC patients in the authors' own database. For the analysis, the Nuclear and Cytoplasmic expression were categorized as high/low on the basis of a threshold of 60% percentage expression. Staging was categorized in low (S1-S2) and high (S3-S4), Grading in high (G3) and low (G1-G2), and Age in old (>= 65 years' old) and non-old (< 65 years' old).

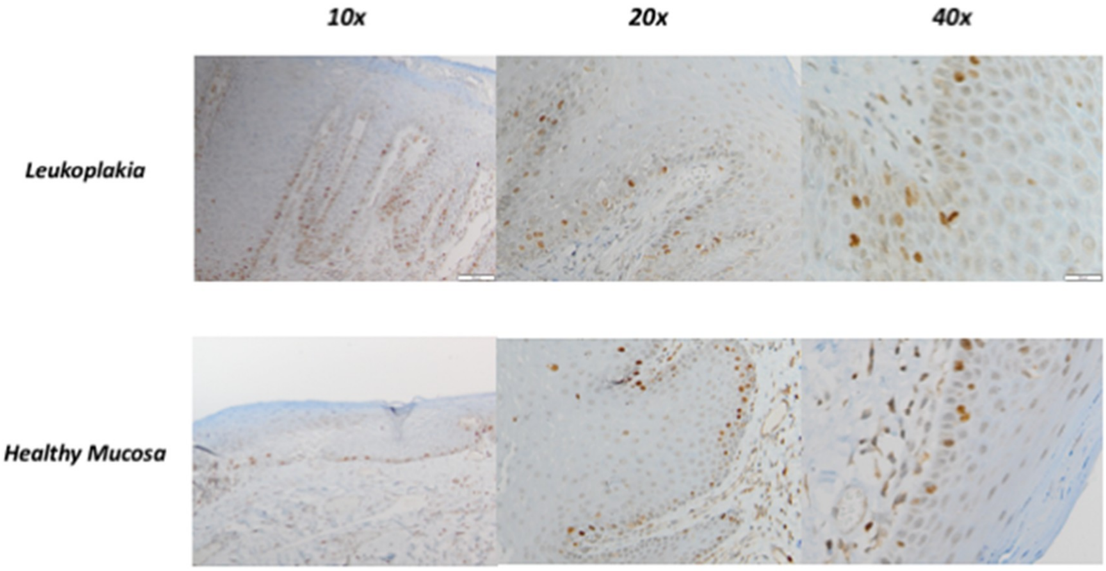

Supplemental Materials: Expression of Survivin in leukoplakia and healthy mucosa samples was mainly nuclear and confined in the basal third of the epithelium.

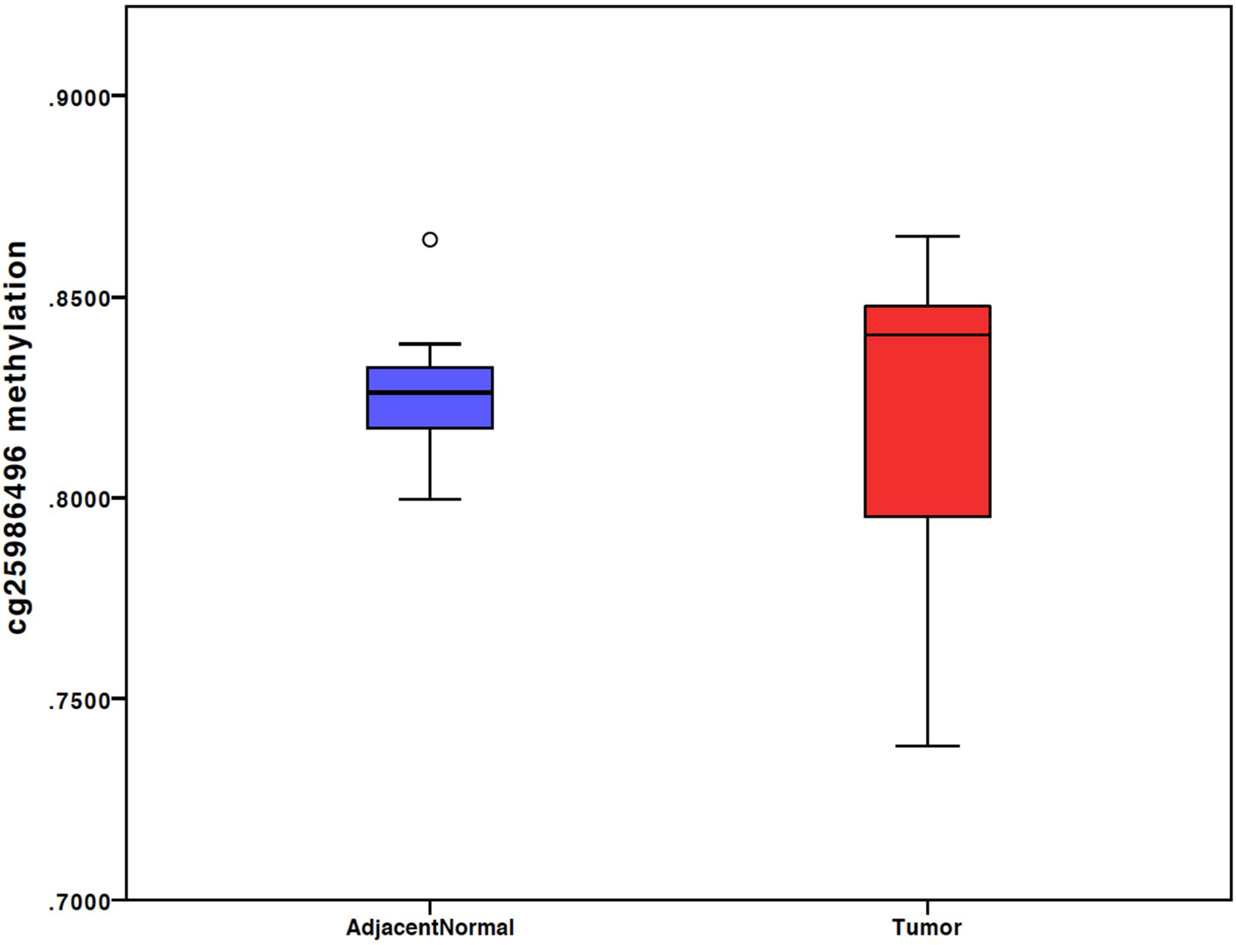

Supplemental Materials: Gene Expression Omnibus (GEO) analysis of methylation of the island cg25986496 in both tumor and paired adjacent normal samples.
